# Active Force Generation in Cardiac Muscle Cells: Mathematical Modeling and Numerical Simulation of the Actin-Myosin Interaction

**DOI:** 10.1007/s10013-020-00433-z

**Published:** 2020-09-02

**Authors:** Francesco Regazzoni, Luca Dedè, Alfio Quarteroni

**Affiliations:** 1 MOX - Dipartimento di Matematica, Politecnico di Milano, P.zza Leonardo da Vinci 32, 20133 Milano, Italy; 2 Mathematics Institute, École Polytechnique Fédérale de Lausanne (EPFL), Av. Piccard, CH-1015 Lausanne, Switzerland

**Keywords:** Mathematical modeling, Cardiac modeling, Active stress, Sarcomeres, Crossbridges, 65M22, 65Z05

## Abstract

Cardiac in silico numerical simulations are based on mathematical models describing the physical processes involved in the heart function. In this review paper, we critically survey biophysically-detailed mathematical models describing the subcellular mechanisms behind the generation of active force, that is the process by which the chemical energy of ATP (adenosine triphosphate) is transformed into mechanical work, thus making the muscle tissue contract. While presenting these models, that feature different levels of biophysical detail, we analyze the trade-off between the accuracy in the description of the subcellular mechanisms and the number of parameters that need to be estimated from experiments. Then, we focus on a generalized version of the classic Huxley model, built on the basis of models available in the literature, that is able to reproduce the main experimental characterizations associated to the time scales typical of a heartbeat—such as the force-velocity relationship and the tissue stiffness in response to small steps—featuring only four independent parameters. Finally, we show how those parameters can be calibrated starting from macroscopic measurements available from experiments.

## Introduction

Cardiovascular diseases represent the worldwide leading causes of death [60], with millions of cases every year. While advancements in medical practice are continuously leading to the development of new therapies and to the improvement of patients care, the role of mathematical and numerical modeling and, more generally, computational medicine, is being increasingly recognized in the context of cardiovascular research. Realistic and accurate in silico models can indeed provide valuable insights on the heart function and support clinicians for personalized treatment of patients [13, 19, 25, 27, 63, 67, 75].

The development of a mathematical and numerical model of the heart function requires integrating together models describing the different physical processes involved, at different spatial scales, in the cardiac activity. The heart is indeed a *multiphysics* and *multiscale* system, whose functions is the result of multiple processes acting in concert to accomplish its main goal, that is pumping blood throughout the body, to supply organs with oxygen and nutrients and to remove the metabolic waste [3, 42, 47, 81]. This process involves an electrophysiological activity (the propagation of an electric potential throughout the cardiac cells membrane and ionic exchanges across the membrane), a subcellular activity (the interactions of contractile proteins) and a mechanical activity (the contraction of the muscle and the resulting blood ejection form the cardiac chambers).

Each process involved in the cardiac function can be described by ad hoc developed mathematical models, written in different forms, including:

- systems of ODEs (Ordinary Differential Equations, see e.g. [1, 7, 35, 38, 52, 61, 68–70, 78, 79, 85]);
- systems of PDEs (Partial Differential Equations, see e.g. [14, 17, 18, 30, 36, 41, 64, 68, 77]);
- continuous-time Markov Chains (see e.g. [39, 72, 76, 82, 83]);
- systems of SDEs (Stochastic Differential Equations, see e.g. [11, 12]).

In this review paper, we focus on the models describing the subcellular processes by which the energy stored in ATP is transformed into mechanical work, thus leading to the contraction of the myocardium. To fulfill their predictive role, these mathematical models should accurately describe the complex mechanisms involved in the process of active force generation. However, very detailed models typically feature large numbers of parameters, which need to be estimated by experimental measurements. The difficulty inherent to direct measures of the subcellular properties of the cardiac tissue calls for a difficult trade-off between the biophysical detail of the models and the identifiability of their parameters.

### Paper Outline

This paper is organized as follows. In Section 2 we illustrate the physiological basis of the active contraction of the cardiac muscle and the main experimental characterizations of this phenomenon, and we highlight the fundamental behaviors that need to be reproduced by mathematical models. Then, in Section 3, we review several mathematical models, available in the literature, describing the mechanisms by which force is generated in the cardiac muscle. In Section 4 we consider the issue of parameter identifiability for force generation models. In particular, we show, for a modified version of the Huxley model [41], how the model parameters can be estimated by measurements typically available from experiments. Finally, in Section 5, we discuss some concluding remarks.

## Active Force Generation in the Cardiac Tissue

**Fig. 1:**
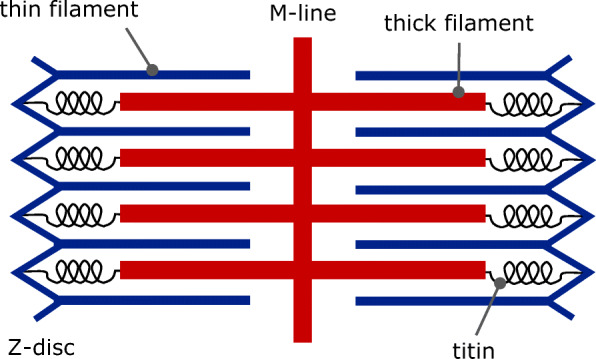
Representation of a sarcomere. Inside sarcomeres, thin and thick filaments are arranged with a regular structure. *M-lines*, located at the center of the sarcomere, have the function of connecting thick filaments together. *Z-discs* link adjacent sarcomeres to each other and to the extracellular matrix and are connected to thick filaments through a huge cytoskeletal protein named *titin*

Sarcomeres, the fundamental contractile units of striated (i.e., skeletal and cardiac) muscles, have a cylindrical shape, with a length ranging from 1.7μm to 2.3μm in physiological conditions. They mainly consist of two types of filaments, thin filaments (or actin filaments, AF) and thick filaments (myosin filaments, MF), arranged in a nearly crystalline structure (see Fig. 1). Active force is generated by the interaction of the protein actin, located in the thin filament, and the protein myosin, located in the thick filaments [3, 42, 47, 81].

The contraction of sarcomeres is triggered by an increase of intracellular calcium ion concentration and can be split into two steps. The first one is the *thin filament regulation*, the second one is the *actomyosin interaction*. The focus of this paper is on the second of the two steps, described in Section 2.1.

In the first step, calcium ions bind to the so-called regulatory units (troponin-tropomyosin complexes located on the thin filaments), thus inducing a conformational change in tropomyosin. Tropomyosin acts as an on-off switch for the actomyosin interaction. When it is in non-permissive state, it *sterically* hinders the binding of myosin with actin binding sites (BSs). This means that the interaction between myosin and actin is prevented because a given tropomyosin protein occupies the BS regulated by that protein. Conversely, when a tropomyosin unit is in permissive state, the regulated actin BSs are free to interact with myosin and to generate force. The actomyosin interaction is a cyclical process, whose simplified view is provided by the *Lymn-Taylor cycle* [53], which is the subject of the next section (Section 2.1).

### The Lymn-Taylor Cycle

Myosin is a molecule made of a coiled–coil tail and two paired heads, capable of binding to actin, thus forming the so-called crossbridges (XBs). Myosin is indeed a molecular motor, which translates the chemical energy stored inside ATP, the primary energy carrier in living organisms, into mechanical work. This is made possible by the so-called *power stroke*, that is a rotation of the attached myosin heads (MHs) which pulls the AF towards the centre of the sarcomere. After the power stroke, the MH detaches and binds to actin in a different position and the cycle is repeated. The joint work of several thousands of pulling MHs makes the sarcomere contract [3, 42, 47, 81].

**Fig. 2:**
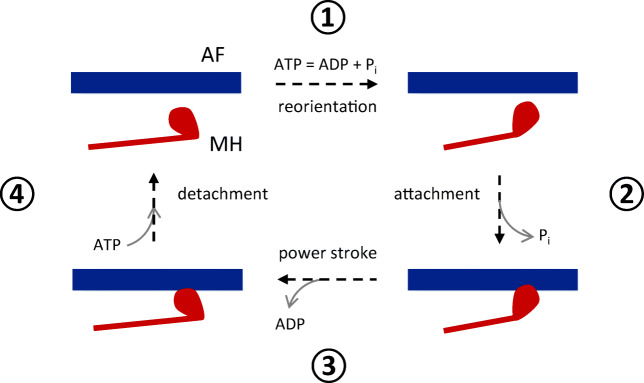
Representation of the Lymn-Taylor cycle

Such attachment-detachment process takes place along a cyclical path. The precise assessment of the functional steps involved in this process is still subject of an active research (see e.g. [8, 37, 59]). A simplified, but sufficient at this level of description, view of it is provided by the so-called Lymn-Taylor cycle (represented in Fig. 2), that comprises the following four steps [3, 11, 48, 53].

1. **ATP hydrolisis.** Myosin, in the stage of the cycle that is traditionally considered as the starting point, is bound to ATP and detached from actin. The catalytic site of myosin hydrolyses ATP into ADP and a phosphate group P_i_ (which remains attached to myosin), transferring to myosin the energy stored in ATP. The MH is still detached from actin, but reoriented and in a higher energetic state.
2. **XB attachment.** The energized MH binds to actin and the phosphate group is released.
3. **Power stroke.** The MH rotates towards the centre of the sarcomere (lower energetic state), thus pulling the actin filament in the same direction. ADP is released from myosin. The force developed by a single power stroke is nearly 0.5–1.0 pN, and the head rotation is nearly 5–10 nm.
4. **XB detachment.** At the end of the power stroke, myosin is tightly bound to actin in a *rigor* configuration, until an ATP molecule binds to myosin, making it detach from actin.

The Lymn–Taylor cycle is repeated, with a pace of nearly five times per second, as long as two conditions are satisfied: enough ATP to fuel the process is available; calcium ion level is high enough to keep tropomyosin in the permissive configuration. When ATP is depleted, the cycle stops in the phase between steps 3 and 4, where all XBs are firmly attached (leading, for skeletal muscle, to the *rigor* state observed in cadavers). When calcium concentration returns to the level corresponding to the relaxed configuration, instead, the cycle is stopped in the phase between steps 1 and 2 [3, 81].

### Force-Velocity Relationship

**Fig. 3:**
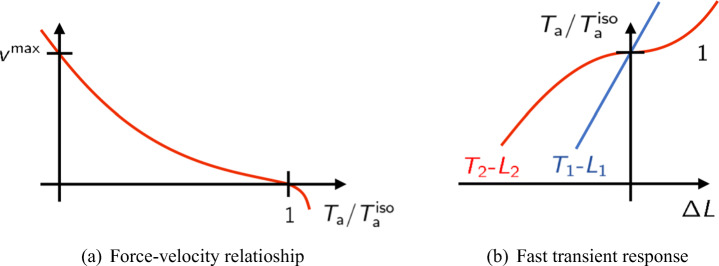
Representation of the force-velocity curve (**a**) and tension-elongation curves after a fast transient (**b**) that is typically obtained in experiments

One of the earliest experimental characterizations of muscle functionality is the force-velocity relationship, dating back to Archibald V. Hill, Nobel Prize winner for his work on the heat production and mechanical work in muscles [31]. In Hill’s experimental setup, a muscle fiber is mounted between a motor and a force transducer. Then, by keeping the muscle in a physiological solution, an electrical stimulus is applied, by keeping constant the fiber length (isometric conditions), until the muscle reaches the steady-state active tension $T_{\text {a}}^{\text {iso}}$. Then the device is switched from the length-control mode to the force-control mode and a negative (or positive) force step is applied. After a transient phase (which is discussed in Section 2.3), the fiber reaches a steady-state with a constant shortening (or lengthening) velocity. The measured force-velocity relationship is a convex curve for positive shortening velocities, connecting the so-called *stall force*, namely the force in isometric conditions ($T_{\text {a}}^{\text {iso}}$), with the *maximum shortening velocity* ($v^{\max \limits }$), in correspondence of which the generated tension is zero (see Fig. 3).

The force in isometric conditions $T_{\text {a}}^{\text {iso}}$ depends on two variables (the sarcomere length *SL* and the calcium concentration inside the cells [Ca^2+^]_i_, where the subscript “i” stands for *intracellular*) that affect the fraction of permissive regulatory units [3, 47]. Clearly, also the force-velocity curves are affected by the same variables; however, when the tension is normalized with respect to its isometric value (i.e., by the steady-state tension obtained for a fixed *SL*), the curves obtained with different values virtually superimpose [3, 9]. This observation suggests that the mechanism underlying the force-velocity relationship is largely independent of the calcium-driven regulation and, therefore, it is linked to the cycling of XBs [9, 11, 48]. The maximum shortening velocity for half-sarcomere is independent on the [Ca^2+^]_i_ and *SL* and it is about $v_{\text {hs}}^{\max \limits } = {8}{\upmu \text {ms}}^{-1}$ (significantly higher than for skeletal muscle).

### Fast Isometric and Isotonic Transients

**Fig. 4:**
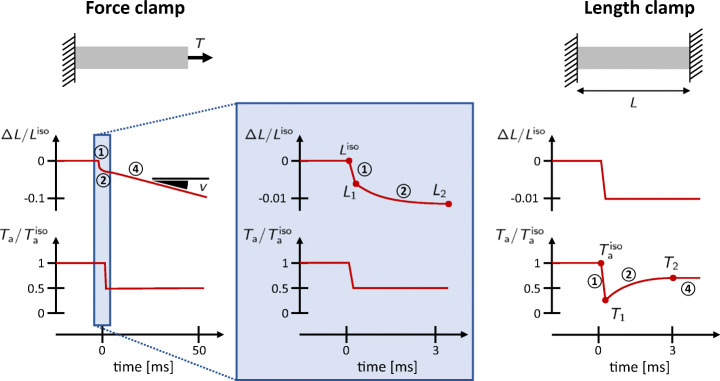
Typical length and tension traces obtained during the *force clamp* (left) and *length-clamp* (right) experiments. In the force clamp case, the slope of the length trace gives the steady-state shortening velocity *v* (represented in the picture). The phases 1,2 and 4 (we recall that phase 3 is absent in the cardiac case) are represented by circled numbers

Fast isometric and isotonic experiments help shedding light on the fastest time scales involved in the dynamics of force generation in the muscle tissue. The two experimental setups are briefly described in what follows (see also Fig. 4).

- **Force clamp** (soft device or isotonic transient). It consists in the same setup employed to obtain the force-velocity relationship. After the isometric force is reached, a step in tension is applied. After a fast transient, the fiber reaches a constant velocity.
- **Length clamp** (hard device or isometric transient). In this case, after that the steady-state is reached while keeping constant the length of the fibers (typically in the range of sarcomere lengths for which the force-length curve is constant, [29, 49, 80]), a step in length is applied (without exiting the above-mentioned plateau region and by keeping the device in length-control mode). The measured force undergoes a fast transient, before returning to the original level.

In both cases, the observed transient can be split into four different phases (even though the third phase is absent in the cardiac tissue), represented in Fig. 4 and associated with different time scales [9, 11, 48, 55, 56].

- **Phase 1** ($\sim {200}{\upmu }$s). In a first phase the tension *T* (respectively, the length of the fiber *L*) changes simultaneously with the step in *L* (respectively, in *T*), until it reaches a level called *T*_1_ (respectively, *L*_1_). Interestingly, by plotting the values of *T*_1_ and *L*_1_ in the *T*-*L* plane, the curves obtained with the soft and hard devices superimpose and show a linear relationship between tension and elongation (Fig. 3(b)). This first phase of the transient is indeed linked to the instantaneous elastic response of XBs. Measurements of the stiffness of this relationship under *rigor* conditions (when the number of attached XBs can be estimated) allow to estimate the stiffness of a single XB [66].
- **Phase 2** ($\sim 2-{3}$ms). After the instantaneous response, tension (respectively, length) quickly reaches a second level, denoted by *T*_2_ (respectively, *L*_2_). Also in this case, the curves of *T*_2_-*L*_2_ obtained with the soft and hard devices superimpose. For lengths close to the rest length, the *T*_2_ tension is very similar to the isometric tension $T_{\text {a}}^{\text {iso}}$, but for larger length steps it is approximately linear in *L*, with a lower stiffness than the elastic stiffness, related to *T*_1_ (Fig. 3(b)). The time scale associated with this phase coincides with the time scale of the power stroke: in this phase, MHs rearrange from the non-equilibrium condition due to the fast step in length until a new equilibrium is reached. Indeed, for small length steps, the power stroke is sufficient for the fibers to almost recover the initial tension level $T_{\text {a}}^{\text {iso}}$.
- **Phase 3 and 4** ($\sim {500}\text {ms}$). After the rapid second phase, in length clamp experiments tension slowly recovers its original level $T_{\text {a}}^{\text {iso}}$ (if the step in length is such that the sarcomeres are still in the plateau region of the force-length relationship). In force clamp experiments, as described in Section 2.2, the filament reaches a steady-state with a constant shortening (or lengthening) velocity. Such velocity, plotted against the isotonic tension, gives the force-velocity curve. This final phase is associated with the XBs attachment and detachment, the slower step of the Lymn-Taylor cycle (see Section 2.1).

Similarly to the force-velocity relationship, when the tension is normalized with respect to the its isometric value, the tension-elongation curves virtually superimpose [9]. This fact supports the hypothesis that the phenomena associated with the fast time scales observed through this experimental setup are linked to the XB dynamics, and not to the regulatory unit dynamics.

## Mathematical Models of the Actomyosin Interaction

In this section, we review several contributions available in the literature to the definition of mathematical models describing the dynamics of XBs. The historical development of such models reflects the progresses in the understanding by the physiologist community of the mechanisms underlying the microscopic force generation. We notice that most of the models are suitable for both the skeletal and the cardiac muscle, provided that the parameters are calibrated accordingly [3, 62].

In this review we limit ourselves to the modeling aspects related to the dynamics of XBs. Moreover, on the basis of the motivations introduced in Section 1, force generation models are here considered in view of multiscale organ-level numerical simulations. This implies that the focus is on those time-scales that are involved in the working regimes occupied by XBs during a heartbeat and that the ultimate goal is predicting the force generated by contracting sarcomeres. In fact, we only partially cover the aspects related to the coupling with the metabolic activity since under physiological conditions it is legitimate to assume that ATP is always available when needed by the myosin motors. For a review on the mathematical models describing the other aspects of muscle contraction (such as the thin filament regulation, the biochemical coupling and the thermodynamic properties of contraction models), we refer the interested reader to [10, 11, 62, 68, 71]. Furthermore, for more details on the multiscale coupling between the microscopic models treated in this section and the macroscopic mechanical behavior of the cardiac tissue, we refer to [63, 67, 70].

### Hill 1938 Model

One of the earliest mathematical descriptions of muscles dates back to [31]. By studying the release of heat when a muscle contracts against a constant load (isotonic contraction), A. V. Hill discovered that the relationship between the active tension *T*_a_ and the shortening velocity *v*_fiber_ is well described by the hyperbolic law:

1 $$ (T_{\text{a}} +a) v_{\text{fiber}}=b_{\text{fiber}} (T_{\text{a}}^{\text{iso}}-T_{\text{a}}), $$

where $T_{\text {a}}^{\text {iso}}$ is the isometric tension (i.e., the tension for *v*_fiber_ = 0), whereas *a* and *b*_fiber_ are positive constants. In what follows, it will be helpful to write relationships that are independent of the length of the muscle fiber used to perform the experiment. With this aim, by dividing (1) by the length of the fiber *L*_fiber_, we get the following relationship:

2 $$ (T_{\text{a}} + a) v = b (T_{\text{a}}^{\text{iso}}-T_{\text{a}}), $$

where we call *v* = *v*_fiber_/*L*_fiber_ the *normalized velocity* (dimensionally, *v* is the inverse of time units). The maximum shortening velocity, that is the maximum speed at which the muscle is able to shorten (see Section 2.2), can be computed as $v^{\max \limits } = b T_{\text {a}}^{\text {iso}} / a$. In the original paper, by fitting the experimental measurements, Hill obtained ${a}/{T_{\text {a}}^{\text {iso}}} = 0.22$, $b_{\text {fiber}} ={1.03}\text {cms}^{-1}$ for a fiber of length *L*_fiber_ = 38mm, thus *b* = 0.27s^− 1^ and $v^{\max \limits } ={1.23}\mathrm {s}^{-1}$ [31].

**Fig. 5:**
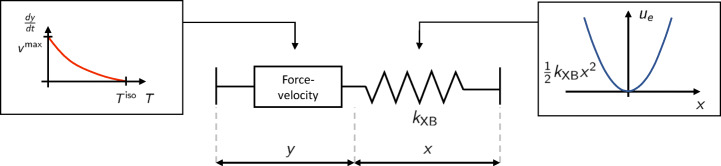
Sketch of the phenomenological model of [31]. A contractile element, following the law (2), is coupled in series with an elastic element, to which a quadratic energy $u_{e}(x) = \frac {1}{2}k x^{2}$ is associated

On the basis of the relationship (2), Hill proposed a phenomenological model where an elastic element is arranged in series with a contractile element governed by the same law (2) (see Fig. 5). This model, however, does not provide any insight into the muscle functioning, as it is not based on a microscopical description of the tissue (this is not surprising since the muscle anatomy was not known at that time).

### Huxley 1957 (H57) Model

**Fig. 6:**
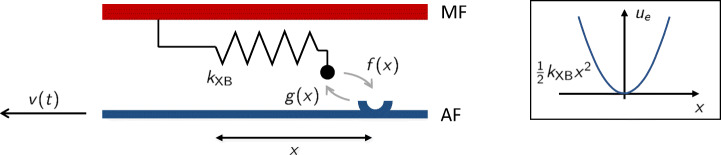
Scheme of the H57 model. The attachment-detachment rates of MHs (denoted respectively by *f* and *g*) depend on the XB distortion *x* (i.e., to which amount the myosin arm is stretched). The myosin arm is modeled as a linear elastic element with stiffness *k*_XB_

In 1957, A.F. Huxley proposed a model (H57 model) to link the force-velocity relationship observed by A.V. Hill with the subcellular attachment-detachment process of MHs [41]. This model considers two states (bound and unbound) and assumes that the transition rates depend on the distance between the myosin arm rest position and the BS, denoted by *x*. We have *x* > 0 when the attachment leads to a positive tension, *x* ≤ 0 otherwise (see Fig. 6).

Let us consider a population of MHs and BSs, and assume that the probability density of finding a couple with a given displacement *x*, denoted by *ρ*_AM_ (actin-myosin pairs density), is constant in an interval sufficiently close to *x* = 0 (more precisely, the number of couples with displacement *x* ∈ (*a*,*b*) for each half filament is *ρ*_AM_|*b* − *a*|, if *a* and *b* are sufficiently close to 0). This is well motivated, assuming the effect of the units located at the border of the filaments negligible.

Let *n*(*x*,*t*) ∈ [0,1] denote the probability that a couple MH-BS with elongation *x* is attached. Then, the expected value of the number of attached XBs with elongation between *a* and *b* at time *t* is given by:

$$ \rho_{\text{AM}} {{\int}_{a}^{b}} n(x,t) dx. $$

Let us consider a small time interval Δ*t*. The variation of the population of attached MHs from *t* to *t* + Δ*t* with displacement in an interval (*a*,*b*) is given (at the first order in Δ*t*) by:

3 $$ \begin{array}{@{}rcl@{}} {{\int}_{a}^{b}} n(x,t+{\Delta} t) dx &\sim& {{\int}_{a}^{b}} n(x,t) dx + n(b,t) v_{\text{hs}}(t) {\Delta} t - n(a,t) v_{\text{hs}}(t) {\Delta} t\\ &&+ {{\int}_{a}^{b}} (1 - n(x,t)) f(x){\Delta} t  dx - {{\int}_{a}^{b}} n(x,t) g(x){\Delta} t  dx, \end{array} $$

where $v_{\text {hs}}(t)= -\frac {d SL(t)/2}{dt}$, the shortening velocity of half sarcomere (that is the relative velocity between the MF and the AF), convects the MH distribution and *f*(*x*) and *g*(*x*) are the attachment and detachment rates, respectively. By dividing the above equation by Δ*t*(*b* − *a*) and letting both intervals go to zero, we get the H57 model:

4 $$  \frac{\partial n (x,t)}{\partial t} - v_{\text{hs}}(t) \frac{\partial n (x,t)}{\partial x} = (1 - n (x,t)) f(x) - n (x,t) g(x), \qquad x \in \mathbb{R},~ t \geq 0, $$

with suitable initial conditions. Finally, assuming that each attached XB acts as a linear spring with stiffness *k*_XB_ (experimentally, *k*_XB_ is of the order of 2pNnm^− 1^), the total force exerted by the pair of interacting half thick filament and thin filament is equal to:

5 $$  F_{\text{hf}}(t) = \rho_{\text{AM}}  k_{\text{XB}} {\int}_{-\infty}^{+\infty}x n(x,t) dx. $$

In [41], the transition rates are phenomenologically set as:

6 $$ f(x) = f_{1} \frac{x}{h}\mathbb{1}_{[0,h]}(x), \qquad g(x) = g_{2}{\mathbb{1}}_{x \leq 0} + g_{1} \frac{x}{h}\mathbb{1}_{x>0}, $$

where *f*_1_, *g*_1_ and *g*_2_ are positive constants. Attachment can occur only in the interval *x* ∈ [0,*h*], that is for positive displacement: such symmetry-breaking feature is what makes the muscle contract. For *x* < 0 the detachment rate is very high, in order to prevent the XBs to generate force in the opposite direction.

The H57 model provides a microscopic explanation of the force-velocity relationship. When the shortening velocity is high, the attached XBs are convected towards lower values of *x*, thus leading to a reduction of force. This mechanism is often compared to a “tug-of-war” game. If the rod is quickly pulled, the players need to detach their hands and reattach them further on the rod, otherwise they are not able to pull any more. Thus, when the rod is sliding towards to players, their action is less efficient than in the steady regime, when they can firmly hold the rod. It is all about how fast the rod slides and how fast the players are in detaching and reattaching their hands. We will see later a quantitative description of the competition between the two phenomena.

With the choice (6), Huxley derived a steady-state solution (with a constant shortening velocity) to (4):

7 $$ n(x) = \begin{cases} \displaystyle F_{1} \left( 1-e^{-\frac{\varphi}{v_{\text{hs}}}}\right)e^{\frac{x}{2h}G_{2}\frac{\varphi}{v_{\text{hs}}}} & \quad x < 0, \\ \displaystyle F_{1} \left( 1-e^{\left( \frac{x^{2}}{h^{2}}-1\right)\frac{\varphi}{v_{\text{hs}}}}\right) & \quad 0 \leq x < h, \\ 0 & \quad x \geq h, \end{cases} $$

where *φ* = (*f*_1_ + *g*_1_)*h*/2, ${F_{1}} = \frac {f_{1}}{f_{1}+g_{1}}$, $G_{2} = \frac {g_{2}}{f_{1}+g_{1}}$. This gives the following force-velocity relationship:

$$ F_{\text{hf}} = \rho_{\text{AM}} k_{\text{XB}} F_{1} \frac{h^{2}}{2} \left( 1 - \frac{v_{\text{hs}}}{\varphi} \left( 1-e^{-\frac{\varphi}{v_{\text{hs}}}}\right) \left( 1 + \frac{1}{2 {G_{2}^{2}}} \frac{v_{\text{hs}}}{\varphi}\right)\right). $$

Huxley, proceeding by trial and error, obtained a good fit of experimental data with *F*_1_ = 13/16 and *G*_2_ = 3.919. For these parameters, by setting *F*_hf_ = 0 we have $v_{\text {hs}}^{\max \limits } \simeq 4 \varphi $. For instance, in [5], with the choice $f_{1} ={65}\mathrm {s}^{-1}$, $g_{1} = {15}\mathrm {s}^{-1}$, $g_{2} ={313.5}\mathrm {s}^{-1}$, *h* = 10nm, one gets $v_{\text {hs}}^{\max \limits } \simeq {1600}\text {nm s}^{-1}$, which gives $v^{\max \limits } = v_{\text {hs}}^{\max \limits } / (SL_{0}/2) \simeq {1.45}\mathrm {s}^{-1}$, were we denote by *SL*_0_ the reference sarcomere length. All the above-mentioned constants are calibrated for the skeletal muscle [48].

We remark that the H57 model, as most of its derivations, focuses on a single pair of half MF and AF. However, because of the three-dimensional arrangement of myofilaments, each MF can possibly bind to six different AFs. In order to account for the actin-myosin binding within the sarcomere lattice, Monte Carlo stochastic models have been proposed (see, e.g. [58]). Nonetheless, the large part of the literature on actin-myosin interactions considers the approximation of focusing on a single pair of myofilaments to be acceptable.

#### The Distribution-Moment Equations

To avoid the solution of a PDE, in [86] an approximation of the model (4) by means of ODEs was proposed. By applying a general strategy of statistical physics, the author computed the equations for the evolution of the distribution-moments of *n*(*x*,*t*), defined as:

$$ \mu^{p}(t) := {\int}_{-\infty}^{+\infty} x^{p} n(x,t) dx. $$

Indeed, thanks to the linear spring hypothesis for the myosin arm, the full distribution *n*(*x*,*t*) is not needed to compute the force, but rather its first moment is enough, as we have, from (5):

$$ F_{\text{hf}}(t) = \rho_{\text{AM}}  k_{\text{XB}}  \mu^{1}(t). $$

By multiplying (4) by *x*^*p*^ and integrating over $(-\infty ,+\infty )$ one gets, for $p = 0,1,\dots $:

8 $$  \frac{d}{d t} \mu^{p}(t) - p v_{\text{hs}}(t)  \mu^{p-1}(t) = {\mu^{p}_{f}} - {\int}_{-\infty}^{+\infty} x^{p} (f(x)+g(x)) n(x,t) dx, $$

where

$$ {\mu^{p}_{f}} := {\int}_{-\infty}^{+\infty} x^{p} f(x) dx $$

denotes the *p* th moment of *f*(*x*) and where we have integrated by parts the term

$$ {\int}_{-\infty}^{+\infty} x^{p} \frac{\partial n (x,t)}{\partial x} dx = \left[x^{p} n(x,t)\right]_{-\infty}^{+\infty} - p {\int}_{-\infty}^{+\infty} x^{p-1} n(x,t) dx = - p\mu^{p-1}(t), $$

and we have used the fact that $n(-\infty ,t) = n(+\infty ,t) = 0$. The last term of (8) needs to be modeled for model closure. In [86] the authors proposed to assume a specific distribution (a gaussian distribution) for *n*(⋅,*t*), so that this term can be computed. Specifically, by assuming that:

$$ n(x,t) = \frac{\mu^{0}(t)}{\sqrt{2\pi}\sigma(t)}\exp\left( -\frac{ (x - \bar{x}(t))^{2}}{2 \sigma^{2}(t)}\right), $$

where

$$ \bar{x}(t) = \frac{\mu^{1}(t)}{\mu^{0}(t)}, \quad \sigma^{2}(t) = \frac{\mu^{2}(t)}{\mu^{0}(t)} -\left( \frac{\mu^{1}(t)}{\mu^{0}(t)}\right)^{2}, $$

the distribution *n*(⋅,*t*) is fully characterized by its first three moments, and thus (8) for *p* = 1,2,3 is completely equivalent to the PDE model (4). However, here we have to pay the price of a strong assumption of gaussianity for *n*(⋅,*t*). In fact, the analytical solution of (7) shows that even in the steady-state case the distribution may be very skewed and thus significantly differ from a gaussian one.

When the transition rates *f*(*x*) and *g*(*x*) take special forms, the distribution-moments strategy can be used to derive exact equivalents to the PDE model (4) [4, 15]. In fact, if the total transition rate is independent of the displacement (i.e. *f*(*x*) + *g*(*x*) = *r*), the last term in (8) can be computed as:

$$ {\int}_{-\infty}^{+\infty} x^{p} (f(x)+g(x)) n(x,t) dx = r \mu^{p}(t), $$

and the hierarchy of (8) can be truncated by considering only the first two moments:

9 $$ \left\{ \begin{array}{rll} \frac{d}{d t} \mu^{0}(t) & = {\mu^{0}_{f}} - r \mu^{0}(t), & \quad t \geq 0, \\ \frac{d}{d t} \mu^{1}(t) & = {\mu^{1}_{f}} - r  \mu^{1}(t) + v_{\text{hs}}(t) \mu^{0}(t),& \quad t \geq 0. \end{array} \right. $$

Similar equations are derived in [20], where the population of MHs is split into three families (pulling, hindering and free MHs) rather than in two families.

#### Extensions of the H57 Model

To account for the fact that not all XBs can be recruitable for attachment (e.g. because a portion of the MF does not face any AF), in [15] the authors modified the source term (1 − *n*(*x*,*t*))*f*(*x*) of (4) into (*n*_0_(*t*) − *n*(*x*,*t*))*f*(*x*), where the reduction factor 0 ≤ *n*_0_(*t*) ≤ 1 denotes the fraction of recruitable XBs.

In [4, 15] the authors introduced a chemical input, affecting the transition rates *f*(*x*) and *g*(*x*), to model the effect of the calcium-driven regulation. Moreover, by assuming that high relative velocities between the two filaments can lead to destruction of XBs, they introduced a further sink term, linearly proportional to |*v*(*t*)|. Specifically, the following transition rates were chosen:

$$ \begin{array}{@{}rcl@{}} f(x,t) &=& k_{\text{ATP}} \mathbb{1}_{x \in [0,1]} \mathbb{1}_{[\text{Ca}^{2+}]_{\text{i}}(t) > C} , \\ g(x,t) &=& k_{\text{ATP}} \mathbb{1}_{x \notin [0,1]} \mathbb{1}_{[\text{Ca}^{2+}]_{\text{i}}(t) > C} + k_{\text{RS}} \mathbb{1}_{[\text{Ca}^{2+}]_{\text{i}}(t) \leq C} + \alpha |v(t)|, \end{array} $$

where *k*_ATP_ is the ATP turnover rate, *C* is the activation threshold for [Ca^2+^]_i_ and *α* is a positive constant. Despite the introduction of the dependence on [Ca^2+^]_i_(*t*) and *v*(*t*), the sum *f*(*x*,*t*) + *g*(*x*,*t*) is still independent of *x*. Hence, distribution-moment equations analogous to (9) can be derived for this model. The thermodynamic properties of this model are assessed in [15].

Velocity-dependent transition rates have been considered in [54] too, where, by assuming that the attachment rate *f*(*x*,*t*) reaches an optimal value for a positive shortening velocity, the authors obtained a better fit of experimental data.

In [51] and [50] the authors proposed a model, based on the H57 formalism, where the population of MHs is split into two pools: the first one contains the MHs located in the single-overlap zone, whereas the other one (for which *f* = 0) contains the remaining MHs. Each pool is characterized by its own density function *n*(*x*,*t*), whose evolution is described by an equation similar to (4), supplemented with a source and a sink term accounting for fluxes across the two pools. Moreover, a variable representing the fraction of permissive BSs multiplies the attachment rate term, in order to account for the calcium-driven force regulation.

#### Limitations of the H57 Model

The models belonging to the family of the H57 model, however, are not able to explain some of the phenomena experimentally observed. In particular, they fail to reproduce the phenomena related to time scales that are faster than the time scale of the power stroke ($\sim {1}\text {ms}$). The reason is that this class of models does not incorporate a description of the power stroke, but rather assumes that MHs attach in a stretched configuration. This cannot explain the fast force recovery following a sudden change in the sarcomere length (see Section 2.3) since, in the H57 model, force is recovered with a time scale that is compatible with the ATP turnover (order of 100ms). These limitations were recognized by A. F. Huxley himself, who proposed, in 1971, a model incorporating an explicit description of the power stroke.

### Power Stroke Models

In [40] the authors proposed a new model (HS71 model), by interpreting the pre-power stroke and the post-power stroke configurations as discrete states. Thus, they introduced a degree of freedom, *y*, that can be interpreted as the angular position of the rotating MH. The variable *y* is associated with a discrete energy potential, with two minima in 0 and *a* (where *a* is the power stroke length), separated by an energy barrier. This newly introduced degree of freedom supplements the linear elastic element of the H57, with potential energy *u*_*e*_(*x*) = *k*_XB_/2(*x* + *y*)^2^.

This *hard-spin* model (i.e., a model where the internal degree of freedom of the MH assumes discrete values) provided a first quantitative description of the power stroke, with the assumption that the fast force recovery (see Section 2.3) is a passive mechanisms, interpretable as a mechanical conformational change. This is coherent with the observation that the fast force recovery is not rate limited by the chemical stages, supporting the hypothesis that the power stroke is a mechanical phenomenon.

The main drawback of the hard-spin HS71 model is that the transition between the two configurations requires the linear spring to be stretched by the effect of thermal fluctuation in order to overcome the energy barrier, as highlighted in [24]. As a consequence, this model predicts a slower time-constant for the power stroke than what is measured in experiments [10, 11]. This led to assume the existence of intermediate configurations, by the introduction of a number of additional states [40, 74]. For instance, in [22], the author proposed a model with strain dependent transition rates and where, thanks to the fast reactions, it is assumed that only three attached states are populated to a sufficient degree. This reduces the parameters of the model to a discrete set, calibrated to reproduce experimentally observed behaviors. A model belonging to the same family, with two detached and three attached states, is proposed in [65].

In [82, 83], the authors considered a full-sarcomere model where the actomyosin interaction is described within the HS71 formalism, that is to say using transitions between discrete states. A continuous variable describing the myosin arm stretch is associated with each MH, so that the transition rates are made dependent on the XB distortion. Due to the complexity of the model, that also includes a description of the regulatory units, its solution is approximated by means of the Monte Carlo method (see e.g. [57]). A similar model, where the crossbridge dynamics is described with a H71-like model, is proposed in [39]. In such models, additional states (besides the two states of the H71 model) are considered.

In 1974, T. L. Hill and coworkers formalized in a unified and thermodynamically sound framework most of the existing attempts to model muscle contraction [32–34]. In their work, they showed that the computation of the generated force requires, in a thermodynamically consistent framework, the knowledge of the energy profiles. On this basis, they proposed in [23] a model where the MH is interpreted as a bistable spring with an internal degree of freedom. However, this degree of freedom is not characterized by a continuous dynamics, but rather its evolution is described as a jump process, with transition rates assigned as phenomenological functions of the strain.

#### Soft-Spin Models

In contrast, in [55, 56] the authors proposed to replace the rigid bistable device (or multi-stable) of hard-spin models by a bistable element, parametrized by a continuous variable. The transition from hard-spin to soft-spin (i.e., the replacement of the discrete internal degree of freedom with a continuous one) removed the contradictions concerning the time scale of the power stroke [11].

This model was extended with the inclusion of the attachment-detachement ATP-driven mechanism by adding a colored (i.e. correlated) noise—mimicking the out-of-equilibrium ATP reactions—to the Langevin dynamics within the energy landscape [56]. The Langevin equation describes the time evolution of a set of degrees of freedom of a molecular system, evolving slowly with respect to other degrees of freedom, featuring a smaller time scale (see e.g. [28]).

This model belongs to the broad family of *Brownian ratchet* models (see e.g. [45]). These models are aimed at explaining how molecular motors can generate motion in a preferred direction when fueled by the energy contained in the surrounding environment. The key ingredients needed to generate motion are: a periodic asymmetric energy landscape, such as the one generated by actin BSs along myofilaments; a colored noise, whose white component represents the heat reservoir surrounding the molecular motor, whereas the correlated one is associated with out-of-equilibrium chemical reactions [11, 16, 43]. One of the first applications of the theory of Brownian ratchets to muscle contraction can be found in [44], with a focus on the attachment-detachment process, rather than on the power stroke.

In [12] the authors proposed a mechano-chemical model (CMC19 model), with a soft-spin model for MHs coupled with a chemical state describing the ATP-driven attachment-detachment process, obtaining a unified framework capable of matching both the phenomena related to the power stroke (such as the fast velocity recovery) and those related to the attachment-detachment of XBs (such as the force-velocity curve). Moreover, the authors showed that the H57 model can be derived from the CMC19 model under simplifying assumptions, thus giving an interpretation to the H57 model in terms of Langevin dynamics. Remarkably, the authors also showed that a lumped version of the CMC19 model in which the power stroke variable is assumed to be in equilibrium formally reduces to a H57-like model, thus allowing to interpret the transition rates of the H57 model as *effective* rates, in light of the CMC19 model. We illustrate in what follows the construction of the CMC19 model.

#### Caruel–Moireau–Chapelle 2019 (CMC19) model

##### Model setup.

**Fig. 7:**
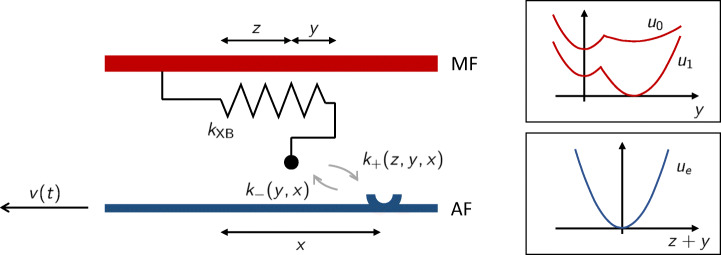
Scheme of the CMC19 model. The MH is described by two degrees of freedom (*z* and *y*). When the MH is attached, the degree of freedom *z* coincides with the variable *x*. The attachment-detachment rates of MHs (*f* and *g*) depend on the XB distortion *x*. The myosin arm is modeled as a linear elastic element with stiffness *k*_XB_, whereas the degree of freedom *y* is associated with a bistable energy, which depends on the XB attachment state

We consider a single MH, described by a discrete degree of freedom, namely *ω*^*t*^ (*ω*^*t*^ = 1 when the MH is attached, *ω*^*t*^ = 0 when it is detached), and two continuous degrees of freedom, namely *Z*^*t*^ (measuring the distance of the MH tip from the rest-position of the myosin arm) and *Y*^*t*^ (associated with the angular orientation of the MH), as it is shown in Fig. 7. In the pre-power stroke configuration, we typically have *Y*^*t*^ = 0, and thus the elongation of the myosin arm coincides with *Z*^*t*^. When power stroke occurs, *Y*^*t*^ becomes positive, making the total myosin arm elogation increase. The myosin arm elogation is indeed given by *Z*^*t*^ + *Y*^*t*^ (see Fig. 7). When the MH is attached (*ω*^*t*^ = 1) the tip of the MH is attached to the BS. Therefore, we have by definition *Z*^*t*^ ≡ *x* (where we denote by *x*, as in the previous sections, the distance between to myosin arm rest position and the BS).

The elastic element is associated with a quadratic energy *u*_*e*_, whereas the internal degree of freedom *Y*^*t*^ is associated with a bistable energy *u*_*ω*_, that has a different expression when the XB is attached and when instead it is not. Specifically, in the attached (respectively, detached) configuration, the minimum corresponding to the post-power stroke configuration (*Y*^*t*^ > 0) is endowed with a lower (respectively, higher) energy than the pre-power stroke configuration (*Y*^*t*^ = 0). The resulting energy landscape for the mechanical variables (*Z*^*t*^,*Y*^*t*^) is thus associated with the energy *w*_*ω*_(*z*,*y*) = *u*_*ω*_(*y*) + *u*_*e*_(*z* + *y*).

The Langevin dynamics (see e.g. [46]) associated with the energy *w*_*ω*_(*z*,*y*) gives the following stochastic differential equation:

10 $$  \left\{ \begin{array}{rll} \eta dZ^{t} =& \left( -\omega^{t}\eta v_{\text{hs}} - (1-\omega^{t})\frac{\partial w_{\omega}}{\partial z}(Z^{t},Y^{t})\right) dt& \\ &+ \eta \delta_{t_{s}}(t) (x - Z^{t}) dt + (1-\omega^{t}) \sqrt{2 \eta k_{B} T} d{B_{z}^{t}}, & \quad t\geq 0,\\ \eta dY^{t} =& -\frac{\partial w_{\omega}}{\partial y}(Z^{t},Y^{t}) dt + \sqrt{2 \eta k_{B} T} d{B_{y}^{t}}, & \quad t\geq 0, \end{array} \right. $$

where $d{B_{z}^{t}}$ and $d{B_{y}^{t}}$ are the increments of a two-dimensional Brownian motion, *η* is the viscous damping coefficient associated with the surrounding fluid, *k*_*B*_ denotes the Boltzmann constant, *T* the absolute temperature, and *t*_*s*_ denotes the time of any switch from *ω*^*t*^ = 0 to *ω*^*t*^ = 1. We notice that, far from *t* = *t*_*s*_, when the XB is detached (i.e., *ω*^*t*^ = 0), the first equation reduces to:

$$ \eta dZ^{t}= -\frac{\partial w_{\omega}}{\partial z}(Z^{t},Y^{t})dt + \sqrt{2 \eta k_{B} T} d{B_{z}^{t}}, $$

while when the XB is attached (i.e., *ω*^*t*^ = 1), it reduces to:

$$ dZ^{t}= -v_{\text{hs}} dt, $$

coherently with the fact that *Z*^*t*^ ≡ *x* (we recall that *v*_hs_ denotes the *shortening* velocity, thus $\dot {x} = -v_{\text {hs}}$). Finally, at time *t* = *t*_*s*_ the Dirac delta term makes the variable *Z*^*t*^ instantaneously jump to *Z*^*t*^ = *x*.

The kinetics of the chemical degree of freedom *ω*^*t*^ is determined by the following transition rates:

$$ \begin{array}{@{}rcl@{}} \mathbb{P}\left[\omega^{t+{\Delta} t}=1|\omega^{t}=0\right] &=& k_{+}(Z^{t},Y^{t},x,t) {\Delta} t + o({\Delta} t), \\ \mathbb{P}\left[\omega^{t+{\Delta} t}=0|\omega^{t}=1\right] &=& k_{-}(Y^{t},x,t) {\Delta} t + o({\Delta} t), \end{array} $$

where the detachment transition rate is independent of *Z*^*t*^ since when the MH is attached we have *Z*^*t*^ = *x*.

##### Fokker–Plank equation.

To write the Fokker–Plank equation (see e.g. [46]) associated with (10), we denote by *p*(*z*,*y*,*ω*;*x*,*t*) the probability density for a MH (at time *t* and located at distance *x*) of being in state (*z*,*y*,*ω*) (we notice that *x* and *t* are regarded as deterministic variables). Since for attached heads we have *Z*^*t*^ = *x*, the probability density for *ω* = 1 can be written as:

$$ p(z,y,1;x,t) = \delta_{x}(z)\bar{p}(y;x,t). $$

With this notation, the Fokker–Plank equation reads:

11 $$ \left\{ \begin{array}{lll} \frac{\partial}{\partial t} p(z,y,0;x,t) =& v_{\text{hs}} \frac{\partial}{\partial x} p(z,y,0;x,t)&\\ &+ \eta^{-1} \frac{\partial}{\partial z} \left( \frac{\partial}{\partial z}w_{0}(z,y)  p(z,y,0;x,t) \right) &\\ &+ \eta^{-1} \frac{\partial}{\partial y} \left( \frac{\partial}{\partial y}w_{0}(z,y)  p(z,y,0;x,t) \right) &\\ &+ \frac{k_{B} T}{\eta} \left( \frac{\partial^{2}}{\partial z^{2}} p(z,y,0;x,t) + \frac{\partial^{2}}{\partial y^{2}} p(z,y,0;x,t) \right)&\\ &+ k_{-}(y,x) \delta_{x}(z) \bar{p}(y;x,t) &\\ &- k_{+}(z,y,x) p(z,y,0;x,t), & x,y,z \in \mathbb{R},~t> 0,\\ {\kern17.5pt}\frac{\partial}{\partial t} \bar{p}(y;x,t) =& v_{\text{hs}} \frac{\partial}{\partial x} \bar{p}(y;x,t)&\\ &+ \eta^{-1} \frac{\partial}{\partial y} \left( \frac{\partial}{\partial y}w_{1}(x,y)  \bar{p}(y;x,t) \right) &\\ &+ \frac{k_{B} T}{\eta} \frac{\partial^{2}}{\partial y^{2}} \bar{p}(y;x,t) &\\ &+ \displaystyle{\int}_{-\infty}^{+\infty} k_{+}(z,y,x) p(z,y,0;x,t) dz& \\ &- k_{-}(y,x) \bar{p}(y;x,t),& x,y \in \mathbb{R},~t> 0, \end{array} \right. $$

endowed with suitable initial conditions. To link this model with the H57 formalism, we notice that the fraction of attached MHs with displacement *x* at time *t* is given by:

$$ n(x,t) = \int\int p(z,y,1;x,t) dz dy = \int \bar{p}(y;x,t) dy. $$

By integrating the equations of (11) with respect to *z* and *y*, we obtain the following H57-like equation:

$$ \frac{\partial n (x,t)}{\partial t} - v_{\text{hs}}(t) \frac{\partial n (x,t)}{\partial x} = (1 - n (x,t)) f(x,t) - n (x,t) g(x,t), $$

where the transition rates are given by:

12 $$ \begin{array}{@{}rcl@{}} f(x,t) &= & \int\int k_{+}(z,y,x) \frac{p(z,y,0;x,t)}{1-n(x,t)} dz dy,\\ g(x,t) &= & \int k_{-}(y,x) \frac{\bar{p}(y;x,t)}{n(x,t)} dy. \end{array} $$

We notice that this H57 version of (11) is not written in closed form, as *f*(*x*,*t*) and *g*(*x*,*t*) depend on the specific distribution of the degrees of freedom *z* and *y* and not only on the averaged quantity *n*(*x*,*t*).

##### Recovering the H57 model.

This analogy with the H57 model allows for a more direct comparison when hypotheses closer to those of the H57 model are assumed. Indeed, by canceling the degree of freedom associated with the power stroke (i.e., *Y*^*t*^ ≡ 0), we have:

$$ \begin{array}{@{}rcl@{}} p(z,y,0;x,t) &=& \hat{p}(z;x,t) \delta(y),\\ \bar{p}(y;x,t) &=& n(x,t) \delta(y), \end{array} $$

which gives, thanks to (12), $g(x,t) = k_{-}(0,x) = \hat {g}(x)$. Moreover, coherently with H57, let us assume that the binding rate is independent of *Z*^*t*^, that is $k_{+}(z,0,x) = \hat {f}(x)$, which gives, thanks to (12), $f(x,t) = \hat {f}(x)$. In this way, in [12], the authors recovered the original H57 model.

##### Thermal equilibrium model.

More interestingly, the authors recovered an analogy with the H57 model under the hypothesis that the time scale of the macroscopic behavior is large enough for the internal degrees of freedom to be at thermal equilibrium. The equilibrium distributions can be multiplicatively decomposed as:

$$ \begin{array}{@{}rcl@{}} p(z,y,0;x,t) &=& p_{0}^{th}(z,y) (1-n(x,t)), \\ \bar{p}(y;x,t) &=& p_{1}^{th}(y;x) n(x,t), \end{array} $$

where

$$ \begin{array}{@{}rcl@{}} p_{0}^{th}(z,y) &=& \frac{\exp\left( -\frac{w_{0}(z,y)}{k_{B} T}\right)}{\int\int\exp\left( -\frac{w_{0}(z,y)}{k_{B} T}\right) dz dy},\\ p_{1}^{th}(y;x) &=& \frac{\exp\left( -\frac{w_{1}(x,y)}{k_{B} T}\right)}{\int\exp\left( -\frac{w_{1}(x,y)}{k_{B} T}\right) dy}. \end{array} $$

When the probability distribution takes this form, (12) reduces to:

13 $$ \begin{array}{@{}rcl@{}} f(x,t) & = & f^{th}(x) = \int\int k_{+}(z,y,x) p_{0}^{th}(z,y) dz dy,\\ g(x,t) & = & g^{th}(x) = \int k_{-}(y,x) p_{1}^{th}(y;x) dy, \end{array} $$

which gives a model, equivalent to the H57 one, in closed form. This conclusion is more than a mere analogy and it allows to shed a new light on the H57 model. The H57 model, which does not explicitly represent the power stroke, can indeed be interpreted as a model where the variable describing the degree of freedom associated with the power stroke is considered at equilibrium. Unlike in the H57 original formulation, where the power stroke is simply neglected, here it is accounted for in the definition of the transition rates given by (13). This allows to relate a microscopic description of the contractile mechanism with macroscopic effective quantities.

## Parameters Estimation in H57-Like Models

In Section 3 we reviewed several models proposed in the literature to describe the dynamics of force generation in the cardiac muscle tissue. Those models feature different levels of biophysical detail in the description of the complex mechanisms that determine active force generation. We have shown how the most detailed models are able to capture phenomena that cannot be captured by the simplest models, such as the fast time scale response of the muscle tissue.

However, when used in specific settings such as in multiscale cardiac simulations (see e.g. [67, 68, 73]), the most detailed models are not necessarily the most suitable to apply. Indeed, some features such as the separation between the phase 1 and phase 2 of fast response (see Section 2.3) cannot be appreciated when the involved time scales are those characterizing the muscle movements during a heartbeat (as we will quantitatively assess later in this section). Moreover, the more detailed a model is, the more parameters need to be calibrated. Because of the difficulty to measure the parameters characterizing the microscopic features of the contractile apparatus, simpler models with fewer parameters (that can be easily calibrated by macroscale measurements) are to be preferred. As a matter of fact, the best compromise between biophysical detail of the model and identifiability of its parameters ought be pursued, by “making things as simple as possible, but not simpler”, to paraphrase a celebrated quote attributed to A. Einstein.

Motivated by the above observations, in this section we consider a generalized version of the H57 model, that encompasses several models available in the literature, to investigate to which extent these models can explain the experimentally observed behaviors linked to the XB dynamics and, at the same time, how the associated parameters can be calibrated by measurements typically available from experiments.

### A Generalized H57 Model

The H57 model is derived under the condition of full activation of the thin filament. To take into account, in a simple way, the fact that not all the regulatory units may be in permissive state (and, thus, the BSs may not be available for XB formation), we consider two options. The first one is to multiply, in the computation of force, the number of XBs by the fraction of permissive BSs, *P*. The second is to replace in (4) the term (1 − *n*(*x*,*t*)) by (*P* − *n*(*x*,*t*)), similarly to what proposed, to account for the filaments overlapping, in [15]. Notice that, thanks to the linearity of the equation, both approaches lead to the same result. Even if this approach is approximate, as it does not take into account the possible time dependence of *P*(*t*), we restrict ourselves to the condition of constant activation.

Hence, we consider the following modified H57 model, where we allow (as in [4, 15]) for a dependency of the transition rate on the shortening velocity *v*_hs_(*t*), and we introduce the dependence on the permissivity *P*:

14 $$ \frac{\partial n (x,t)}{\partial t} - v_{\text{hs}}(t) \frac{\partial n (x,t)}{\partial x} = (P - n (x,t)) f(x,v(t)) - n (x,t) g(x,v(t)), \quad x \in \mathbb{R},~t \geq 0, $$

where we prefer to express the transition rates in function of the normalized shortening velocity *v*(*t*) = *v*_hs_(*t*)/(*SL*_0_/2). The force generated by half filament, by assuming that a XB attached with displacement *x* exerts a force *F*_XB_(*x*), is given by:

$$ F_{\text{hf}}(t) = \rho_{\text{AM}} {\int}_{-\infty}^{+\infty} F_{\text{XB}}(x) n(x,t) dx. $$

In particular, with a linear spring XB model (i.e., *F*_XB_(*x*) = *k*_XB_*x*), we have:

15 $$  F_{\text{hf}}(t) = \rho_{\text{AM}} k_{\text{XB}} {\int}_{-\infty}^{+\infty} x  n(x,t) dx. $$

The macroscopic tension, in turn, is proportional to the force generated by half filament. We remark that (14) should not be regarded as a new model, but rather as a general formulation encompassing several models available in the literature, thus allowing to analyze them in a unified framework.

In (14), the quantities to be modeled (that is the “parameters” of the model) are *f*(*x*,*v*) and *g*(*x*,*v*). Clearly, without a detailed microscopic model of the attachment-detachment process, the two functions *f*(*x*,*v*) and *g*(*x*,*v*) cannot be easily calibrated from macroscale experiments.

### Distribution-Moments Equation

Under the hypothesis that the total transition rate is independent of *x* (i.e., there exists a function *r*(*v*) = *f*(*x*,*v*) + *g*(*x*,*v*)), it is possible to write the distribution-moments equations (see Section 3.2). With this aim, we introduce the moments for $p \in \mathbb {N}$ (we notice that, differently from the notation used in Section 3.2, *μ*^*p*^ are dimensionless, whereas ${\mu _{f}^{p}}$ are inverse of time units):

16 $$ \begin{array}{@{}rcl@{}} \mu^{p}(t) & := & {\int}_{-\infty}^{+\infty} \left( \frac{x}{SL_{0}/2}\right)^{p} n(x,t) \frac{dx}{D_{M}},\\ {\mu_{f}^{p}}(v) & := & {\int}_{-\infty}^{+\infty} \left( \frac{x}{SL_{0}/2}\right)^{p} f(x,v) \frac{dx}{D_{M}}. \end{array} $$

Thanks to the normalization by *D*_*M*_ (i.e., the distance between two consecutive MHs along the thick filament, corresponding to nearly 43nm, see e.g. [3]), *μ*^0^(*t*) can be interpreted as the fraction of BSs involved in a XB. Moreover, *μ*^1^(*t*)/*μ*^0^(*t*) corresponds to the average distortion of attached XBs, normalized with respect to *SL*_0_/2. We notice that, under the linear spring hypothesis, thanks to (15), the total active tension is proportional to *μ*^1^(*t*). Therefore, we can write *T*_a_(*t*) = *a*_XB_*μ*^1^(*t*), where *a*_XB_ has the dimension of a pressure. More precisely, *a*_XB_ is a proportionality constant that allows to upscale the microscopically generated force to the macroscopic stress of the tissue. As a matter of fact, *a*_XB_ is proportional to the XB stiffness *k*_XB_ and to the filament area density in the cross-filament section of the muscle tissue.

By multiplying (14) by (*x*/(*SL*_0_/2))^*p*^, integrating over $x \in (-\infty , + \infty )$ and using the fact that $n(-\infty ,t) = n(+\infty ,t) = 0$, we get the following distribution-moments equations:

17 $$ \left\{ \begin{array}{rll} \frac{d}{dt}{\mu^{0}}(t) &= - r(v(t)) \mu^{0}(t) + P{\mu_{f}^{0}}(v(t)),& \quad t\geq 0, \\ \frac{d}{dt}{\mu^{1}}(t) &= - r(v(t)) \mu^{1}(t) + P{\mu_{f}^{1}}(v(t)) - \mu^{0}(t) v(t),& \quad t\geq 0. \end{array} \right. $$

By assuming that *f* + *g* is independent of *x*, the freedom in the choice of the functions describing the model has been reduced, as we have to model ${\mu _{f}^{0}}(v)$, ${\mu _{f}^{1}}(v)$ and *r*(*v*), that are function of *v* only.

### Steady-State Solution

By assuming a constant shortening $v(t) \equiv \bar {v}$, and solving (17) by setting all time derivatives equal to zero, we get the following steady-state solution:

18 $$ \begin{array}{ll} \bar{\mu}^{0} &= P \frac{{\mu_{f}^{0}}(\bar{v})}{r(\bar{v})},\\ \bar{\mu}^{1} &= P \frac{{\mu_{f}^{1}}(\bar{v}) - \mu^{0}(t) \bar{v}}{r(\bar{v})} = P\left( \frac{{\mu_{f}^{1}}(\bar{v})}{r(\bar{v})} - \frac{{\mu_{f}^{0}}(\bar{v})}{r(\bar{v})^{2}} \bar{v} \right). \end{array} $$

Since the force is proportional to *μ*^1^, the last equation gives the force-velocity relationship. Moreover, the steady-state solution of (18) allows to compute some quantities of interest. The force in isometric conditions is given by $T_{\text {a}}^{\text {iso}} = a_{\text {XB}} (\bar {\mu }^{1})_{\bar {v} = 0} = a_{\text {XB}} P{\mu _{f}^{1}}(0)/r(0)$. The fraction of attached XBs, in turn, is given by $(\bar {\mu }^{0})_{\bar {v} = 0} = P {\mu _{f}^{0}}(0)/r(0)$. Finally, the maximum shortening velocity $v^{\max \limits }$ can be computed as the positive solution of the equation ${\mu _{f}^{1}}(v^{\max \limits })r(v^{\max \limits }) = {\mu _{f}^{0}}(v^{\max \limits })v^{\max \limits }$.

As a matter of fact, the above-mentioned quantities take special forms under more restrictive hypotheses for *f* and *g*. For instance, it is reasonable to assume that the sliding velocity only affects the detachment rate, so that $f(x,v) = \bar {f}(x)$. In this case, assuming again that the sum *f* + *g* is independent of *x*, we can write $g(x,v) = r_{0} - \bar {f}(x) + q(v)$, for some *q*(*v*) such that *q*(0) = 0 and where *r*_0_ = *r*(0). The term *q*(*v*) models the rate of XB destruction due to rapid length changes. Under this additional hypothesis, the objects to be modeled are just $\mu _{\bar {f}}^{0}$, $\mu _{\bar {f}}^{1}$, *r*_0_ and *q*(*v*) (three scalar values and a function). If we set, as in [15], *q*(*v*) = *α*|*v*| (which reduces the quantities to be modeled to 4 scalars), the maximum shortening velocity takes the form:

$$ v^{\max} = {r_{0}}\left( \frac{\mu_{\bar{f}}^{0}}{\mu_{\bar{f}}^{1}} -\alpha\right)^{-1}. $$

Let us consider now the particular case of constant attachment rate within the interval *x* ∈ [*s*_0_,*s*_0_ + *h*] (as in [4]):

$$ f(x,v) = k_{\text{ATP}} \mathbb{1}_{[s_{0},s_{0}+h]}(x), \qquad g(x,v) = k_{\text{ATP}} \left( 1-\mathbb{1}_{[s_{0},s_{0}+h]}(x)\right) + q(v). $$

This choice falls within the above-mentioned case (i.e., the sum *f* + *g* is independent of *x* and *v* affects only *g*). In particular, we have *r*_0_ = *k*_ATP_. The quantities to be modeled, in this case, are *k*_ATP_, *h*, *s*_0_, *q*(*v*), which are linked to the previous ones by:

$$ \mu_{\bar{f}}^{0} = k_{\text{ATP}} \frac{h}{D_{M}}, \qquad \mu_{\bar{f}}^{1} = k_{\text{ATP}} \frac{h(h + 2 s_{0})}{SL_{0}  D_{M}}, \qquad r_{0} = k_{\text{ATP}}, $$

and, conversely:

$$ h = \frac{k_{\text{ATP}}}{\mu_{\bar{f}}^{0} D_{M}}, \qquad s_{0} = \frac{1}{2}\left( \frac{SL_{0}  D_{M} \mu_{\bar{f}}^{1}}{k_{\text{ATP}} h} - h\right), \qquad k_{\text{ATP}} = r_{0}, $$

which allows to give a microscopical interpretation to the constants. In this case, the steady-state solution reads:

$$ \begin{array}{@{}rcl@{}} \bar{\mu}^{0} &=& P \frac{h}{D_{M}}\left( 1 + \frac{q(\bar{v})}{k_{\text{ATP}}}\right)^{-1},\\ \bar{\mu}^{1} &=& P \frac{h}{2  D_{M}} \left( 1 + \frac{q(\bar{v})}{k_{\text{ATP}}}\right)^{-2} \left( 2\frac{h + 2 s_{0}}{SL_{0}} \left( 1 + \frac{q(\bar{v})}{k_{\text{ATP}}}\right) - 2 \frac{\bar{v}}{k_{\text{ATP}}}\right). \end{array} $$

Moreover, the isometric tension is given by $T_{\text {a}}^{\text {iso}} = a_{\text {XB}} P \frac {h(h + 2 s_{0})}{SL_{0} D_{M}}$ and the fraction of attached XBs in isometric conditions is $(\bar {\mu }^{0})_{\bar {v} = 0} = P\frac {h}{D_{M}}$. With the choice *q*(*v*) = *α*|*v*|, the maximum shortening velocity, if $\alpha < \frac {SL_{0}}{h + 2 s_{0}}$, is given by:

$$ v^{\max} = k_{\text{ATP}}\left( \frac{SL_{0}}{h + 2 s_{0}} -\alpha\right)^{-1}. $$

Conversely, if $\alpha \geq \frac {SL_{0}}{h + 2 s_{0}}$, $v^{\max \limits }$ is not defined, as the force-velocity relationship never intercepts the *T*_a_ = 0 axis.

### Fast Transient Solution

Because of the lack of explicit representation of the power stroke, the generalized H57 model (16) fails to reproduce the three different phases after a fast step, either in length or in tension (see Section 2.3). Indeed, in place of the two fast steps (the elastic response and the fast force recovery, due to the power stroke), we have only a single fast step, followed by the slow force recovery (or by the constant shortening, in the case of the soft device experiment). In this section, we study the predictions of the model concerning such a phase.

In order to study the behavior predicted by the model when a fast transient experiment is performed (here we focus on steps in length), we suppose that at *t* = 0 the muscle is in steady-state isometric conditions (i.e., $\mu ^{0}(0) =P {\mu _{f}^{0}}(0)/r(0)$, $\mu ^{1}(0) = P {\mu _{f}^{1}}(0)/r(0)$). We then consider a sudden change in length Δ*L* (the relative shortening w.r.t. half sarcomere, thus a dimensionless quantity), accomplished in a small amount of time *δ* (i.e., $v(t) = \frac {\Delta L}{\delta } \mathbb {1}_{[0,\delta ]}(t)$). We study the solution at *t* = *δ*, for *δ* → 0^+^.

The solution of (17), when $v(t) = \bar {v}$ is constant, is given by:

19 $$ \left\{ \begin{array}{rll} \mu^{0}(t) =& \mu^{0}(0) + \left( P \frac{{\mu_{f}^{0}}(\bar{v})}{r(\bar{v})} - \mu^{0}(0)\right) \left( 1-e^{-r(\bar{v})t}\right),& \quad t\geq 0,\\ \mu^{1}(t) =& \mu^{1}(0) + \left( P\left( \frac{{\mu_{f}^{1}}(\bar{v})}{r(\bar{v})} - \frac{{\mu_{f}^{0}}(\bar{v})}{r(\bar{v})^{2}} \bar{v} \right) - \mu^{1}(0)\right) \left( 1-e^{-r(\bar{v})t}\right)\\ &+\left( P\frac{{\mu_{f}^{0}}(\bar{v})}{r(\bar{v})} - \mu^{0}(0)\right)\bar{v} t e^{-r(\bar{v})t},& \quad t\geq 0. \end{array} \right. $$

By setting $\bar {v} = \frac {\Delta L}{\delta }$, the tension at the end of the length step reads:

20 $$ \begin{array}{@{}rcl@{}} T_{\text{a}}(\delta) = a_{\text{XB}}\mu^{1}(\delta) &=& a_{\text{XB}}P\left[\frac{{\mu_{f}^{1}}}{r(0)} + \left( {\mu_{f}^{1}} \left( \frac{1}{r(\bar{v})} - \frac{1}{r(0)}\right) - \frac{{\mu_{f}^{0}}}{r(\bar{v})^{2}} \frac{\Delta L}{\delta} \right)\left( 1-e^{-r(\bar{v})\delta}\right)\right.\\ &&\left. + {\mu_{f}^{0}} \left( \frac{1}{r(\bar{v})} - \frac{1}{r(0)}\right){\Delta} L e^{-r(\bar{v})\delta}\right]. \end{array} $$

For time *t* > *δ*, the solution is given by (19), shifted by *δ*, with $\bar {v} = 0$ and with initial state given by (20). However, to characterize the fast phase, we are here only interested in studying the asymptotic behavior of (20) for *δ* → 0^+^. The solution depends on the behavior of *r*(*v*) for $v \to +\infty $. We distinguish between four possible cases: bounded or with sublinear, linear or superlinear growth.

- **Saturating behavior.** Suppose that for $v \to +\infty $, $r(v) \to r_{\max \limits }$. Then, we have:
  $$ \begin{array}{@{}rcl@{}} T_{\text{a}}(\delta) &\sim& a_{\text{XB}} P \left[\frac{{\mu_{f}^{1}}}{r(0)} - \frac{{\mu_{f}^{0}}}{r_{\max}} {\Delta} L + {\mu_{f}^{0}} \left( \frac{1}{r_{\max}} - \frac{1}{r(0)}\right){\Delta} L \right]\\ &=& \frac{a_{\text{XB}}P{\mu_{f}^{1}}}{r(0)} - \frac{a_{\text{XB}}P{\mu_{f}^{0}}}{r(0)}{\Delta} L, \end{array} $$
  which is a linear response, with slope $\frac {a_{\text {XB}}P{\mu _{f}^{0}}}{r(0)}$. In this case, therefore, the fast response is that of a linear elastic spring (like the *T*_1_-*L*_1_ curve), with stiffness given by $\frac {a_{\text {XB}}P{\mu _{f}^{0}}}{r(0)}$.
- **Sublinear growth.** Suppose that $r(v) \to +\infty $, but *r*(*v*)/*v* → 0. Then we have $r(\bar {v})\delta = r(\frac {\Delta L}{\delta })\delta \to 0$, and thus:
  $$ T_{\text{a}}(\delta) \sim \frac{a_{\text{XB}}P{\mu_{f}^{1}}}{r(0)} - \frac{a_{\text{XB}}P{\mu_{f}^{0}}}{r(0)}{\Delta} L, $$
  which is the same behavior as the previous case. For this reason, from now on, we will include both cases in the sublinear growth one.
- **Linear growth.** Suppose now that $r(v) \sim \alpha v$. In this case, we have $r(\bar {v})\delta = r(\frac {\Delta L}{\delta })\delta \sim \alpha {\Delta } L $ and thus:
  $$ T_{\text{a}}(\delta) \sim \frac{a_{\text{XB}}P{\mu_{f}^{1}}}{r(0)} e^{-\alpha {\Delta} L} - \frac{a_{\text{XB}}P{\mu_{f}^{0}}}{r(0)}e^{-\alpha {\Delta} L}{\Delta} L. $$
  Hence, in this case the response is different from a linearly elastic element. In order to compare the stiffness for small step lengths with the stiffness predicted in the sublinear growth case, we linearize around Δ*L* = 0, getting:
  $$ T_{\text{a}}(\delta) \sim \frac{a_{\text{XB}}P{\mu_{f}^{1}}}{r(0)} - a_{\text{XB}}P\frac{{\mu_{f}^{0}} + \alpha {\mu_{f}^{1}}}{r(0)}{\Delta} L. $$
  In conclusion, the stiffness associated with small steps is increased by a term $\alpha a_{\text {XB}} P {\mu _{f}^{1}}/r(0)$.
- **Superlinear growth.** Suppose that $r(v) \to +\infty $ and $r(v)/v \to +\infty $. Then we have $r(\bar {v})\delta = r(\frac {\Delta L}{\delta })\delta \to +\infty $, which gives:
  $$ T_{\text{a}}(\delta) \to 0. $$
  This means that, if the destruction rate grows more than linearly in the velocity, then, in the limit of an instantaneous length step, the velocity is such that all the XB are destructed.

### Parameter Calibration

As noticed in Section 4, the calibration of the generalized H57 model (16) requires the definition of the functions *f*(*x*,*v*) and *g*(*x*,*v*). However, such functions, without a detailed microscopical model, are difficult to be determined solely based on experimental results. By assuming that the sum *f* + *g* is independent of *x* and that *v* only affects detachment, instead, the objects to be estimated reduce to the four scalars $\mu _{\bar {f}}^{0}$, $\mu _{\bar {f}}^{1}$, *r*_0_, *a*_XB_ and the function *q*(*v*). In addition, as shown in Section 4.4, the response to fast transients is only affected by the asymptotic behavior of *q*(*v*) for $|v| \to +\infty $, whereas the force-velocity relationship is only affected by the values of *q*(*v*) for $0 \leq v \leq v^{\max \limits }$. Therefore, in what follows, we will restrict ourselves to the following two cases:

- **Sublinear growth**: we consider *q*(*v*) such that *q*(*v*) = *α*|*v*| for small velocities, whereas for $|v| \to +\infty $ we have *q*(*v*)/|*v*|→ 0.
- **Linear growth**: we consider for simplicity the case *q*(*v*) = *α*|*v*|.

We do not consider the case of superlinear growth since in the limit of instantaneous response it predicts the detachment of all the XBs, which hinders the possibility of fitting any fast response curve.

The behavior of the model is thus determined by five scalar parameters ($\mu _{\bar {f}}^{0}$, $\mu _{\bar {f}}^{1}$, *r*_0_, *a*_XB_, *α*) and by the asymptotic of behavior *q*(*v*) (linear or sublinear). This entails a significantly enhanced identifiability of the model parameters with respect to the case when one needs to determine the functions *f*(*x*,*v*) and *g*(*x*,*v*), that are infinite dimensional objects. The possibility of uniquely determine the parameters of a model from the available experimental measurements is of fundamental importance for the predictivity of the model itself.

As a matter of fact, from the previous sections, it follows that by acting on the above-mentioned parameters, the generalized H57 model can match the following experimentally measured quantities.

**Fig. 8:**
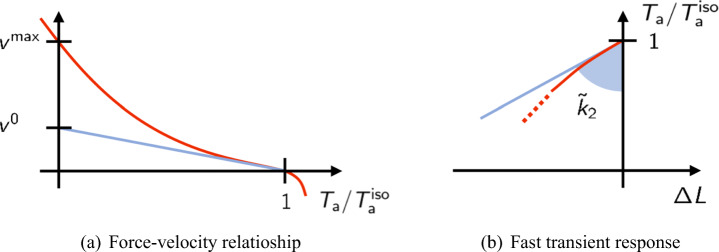
The force-velocity relationship (a) is characterized by the maximum shortening velocity $v^{\max \limits }$ (the intercept of the curve with the axis *T*_a_ = 0) and by the inverse sensitivity of the force to velocity in isometric conditions *v*^0^, which can be interpreted as the intercept with the axis *T*_a_ = 0 of the tangent to the curve in isometric conditions. On the other hand, the response to fast transients is characterized by the normalized stiffness $\tilde {k}_{2}$, where the subscript 2 reflects the fact that this value characterizes the *T*_2_-*L*_2_ response

- Under **isometric conditions**, the solution allows to compute the following quantities.
  - The isometric tension:
    $$ T_{\text{a}}^{\text{iso}} = a_{\text{XB}} (\bar{\mu}^{1})_{\bar{v} = 0} = a_{\text{XB}} P\frac{\mu_{\bar{f}}^{1}}{r_{0}}. $$
  - The fraction of attached XBs:
    $$ \mu_{\text{iso}}^{0} := (\bar{\mu}^{0})_{\bar{v} = 0} = P\frac{\mu_{\bar{f}}^{0}}{r_{0}}. $$
- The **force-velocity** is invariant after normalization with respect to the isometric tension (see Section 2.2). The generalized H57 model correctly predicts this fact. If we suppose, for instance, to vary the calcium concentration and consequently the value of *P*, the normalized force-length relationship would be unaffected. Indeed, the normalized force-length relationship is given by:
  $$ T_{\text{a}}/T_{\text{a}}^{\text{iso}} = \frac{1}{1+\alpha \frac{|v|}{r_{0}}} - \frac{\mu_{\bar{f}}^{0} / \mu_{\bar{f}}^{1}}{\left( 1+\alpha \frac{|v|}{r_{0}}\right)^{2}} \frac{v}{r_{0}}. $$
  Unlike the original H57 model, that predicts a linear force-velocity relationship (corresponding to the case *α* = 0), by allowing for a dependence of the detachment rate on the velocity, the experimentally observed convex shape can be obtained. Indeed, by properly choosing the parameters of the model, one can fit the following two quantities, characterizing the relationship for large and for small velocities, respectively.
  - ◦ The maximum shortening velocity:
    $$ v^{\max} = {r_{0}}\left( \frac{\mu_{\bar{f}}^{0}}{\mu_{\bar{f}}^{1}} -\alpha\right)^{-1}. $$
  - ◦ The inverse of the sensitivity of the normalized force w.r.t. velocity changes in isometric conditions (whose interpretation in the force-velocity curve is shown in Fig. 8(a)):
    $$ v^{0} := - \left.\left( \frac{\partial \bar{T}_{\text{a}} / T_{\text{a}}^{\text{iso}}}{\partial v} \right|_{v = 0}\right)^{-1} = r_{0}\left( \frac{\mu_{\bar{f}}^{0}}{\mu_{\bar{f}}^{1}} +\alpha\right)^{-1}. $$
  With the original H57 model, having *α* = 0, we have $v^{\max \limits } = v^{0}$ and the behaviors at small and large velocities cannot be decoupled.
- The **fast transient** response is characterized by two distinct curves, associated with different time scales (see Section 2.3). As previously noticed, models belonging to the H57 class do not incorporate a description of the power stroke and are thus only capable of reproducing the instantaneous linear response. However, if we interpret the H57 model as the limit of a more detailed model where the power stroke is considered at equilibrium (see Section 3.3.2), the fast response is only characterized by a single time constant, corresponding to the slowest of the two time constants observed experimentally. Such a time constant, therefore, corresponds to the second of the phases considered in Section 2.3. For this reason, we interpret the stiffness associated with fast steps in the generalized H57 model of (16) as the stiffness associated with the *T*_2_-*L*_2_ curve. In particular, the parameters can be chosen so that the following values are fitted.
  - ◦ The tangent normalized stiffness in isometric conditions (see Fig. 8(b)):
    $$ \tilde{k}_{2} := - \left.\frac{\partial T_{\text{a}}(0^{+}) / T_{\text{a}}^{\text{iso}} }{\partial {\Delta} L}\right|_{\Delta L = 0} = \left\{\begin{array}{ll} \mu_{\bar{f}}^{0}/\mu_{\bar{f}}^{1} & \text{sublinear q}, \\ \mu_{\bar{f}}^{0}/\mu_{\bar{f}}^{1} + \alpha & \text{linear q}. \end{array}\right. $$
  Moreover, we notice that, if one is only interested in macroscopic regimes characterized by sufficiently large time scales, only the region of the *T*_2_-*L*_2_ curve associated with small steps is of interest. Indeed, the larger the length step is, the higher shortening velocities are needed to appreciate the distinction between phase 2 and phases 3–4 of the response (we will quantitatively support this point in Section 4.6). In conclusion, since in the region associated with small steps a linear fit provides a good approximation of the curve, the quantity $\tilde {k}_{2}$ alone provides a sufficiently complete characterization of the fast step response.

The five parameters characterizing the generalized H57 model (16) can be assigned to match the five measured quantities $T_{\text {a}}^{\text {iso}}$, $\mu ^{0}_{\text {iso}}$, $v^{\max \limits }$, *v*^0^ and $\tilde {k}_{2}$. This provides a practical way of calibrating the model parameters from experimental measurements. Specifically, in the linear growth case, the parameters of the model can be determined by the following relationships:

21 $$ \begin{array}{@{}rcl@{}} r_{0} &=& \tilde{k}_{2}  v^{0},\\ \alpha &=& \frac{r_{0}}{2}\left( (v^{0})^{-1} - (v^{\max})^{-1}\right) = \frac{\tilde{k}_{2}}{2} \left( 1 - \frac{v^{0}}{ v^{\max}}\right),\\ \mu_{\bar{f}}^{0} &=& \frac{\mu^{0}_{\text{iso}} r_{0}}{P} = \frac{\mu^{0}_{\text{iso}} \tilde{k}_{2} v^{0}}{P},\\ \mu_{\bar{f}}^{1} &=& \left( \tilde{k}_{2} - \alpha\right)^{-1} \mu_{\bar{f}}^{0},\\ a_{\text{XB}}&=& \frac{T_{\text{a}}^{\text{iso}} r_{0}}{\mu_{\bar{f}}^{1} P} = \frac{T_{\text{a}}^{\text{iso}}\tilde{k}_{2} (1 + \frac{v^{0}}{v^{\max}})}{2\mu^{0}_{\text{iso}}}. \end{array} $$

Conversely, in the sublinear growth case we have:

22 $$ \begin{array}{@{}rcl@{}} r_{0} &=& \frac{2  \tilde{k}_{2} v^{\max}}{1 + v^{\max}/v^{0}},\\ \alpha &=& \frac{ v^{\max}-v^{0}}{ v^{\max}+v^{0}}\tilde{k}_{2},\\ \mu_{\bar{f}}^{0} &=& \frac{\mu^{0}_{\text{iso}} r_{0}}{P} ,\\ \mu_{\bar{f}}^{1} &=& \mu_{\bar{f}}^{0} / \tilde{k}_{2},\\ a_{\text{XB}}&=& \frac{T_{\text{a}}^{\text{iso}} r_{0}}{\mu_{\bar{f}}^{1} P}. \end{array} $$

In both cases of linear and sublinear growth, *P* denotes the permissivity associated with the condition in which $T_{\text {a}}^{\text {iso}}$ and $\mu ^{0}_{\text {iso}}$ are measured.

#### *Remark 1*

Among the five quantities used to calibrate the model parameters, only one (namely $\mu ^{0}_{\text {iso}}$) is related to the microscopic scale, whereas the others are related to the macroscale. The measurement of $\mu ^{0}_{\text {iso}}$ may be hard to be accomplished, indeed. However, if one is interested only in the prediction of the generated tension and not in the moments *μ*^0^ and *μ*^1^, the calibration can be accomplished regardless of $\mu ^{0}_{\text {iso}}$, by considering only the macroscopic scale. As a matter of fact, the three parameters *a*_XB_, $\mu _{\bar {f}}^{0}$ and $\mu _{\bar {f}}^{1}$ appear always in the two combinations $a_{\text {XB}} \mu _{\bar {f}}^{1}$ and $\mu _{\bar {f}}^{0}/\mu _{\bar {f}}^{1}$, apart from the expression of $\mu ^{0}_{\text {iso}}$. Therefore, one could calibrate the two combined terms $a_{\text {XB}}\mu _{\bar {f}}^{1}$ and $\mu _{\bar {f}}^{0}/\mu _{\bar {f}}^{1}$ rather than the three parameters. In other terms, thanks to the linearity of the equations, the value of $\mu ^{0}_{\text {iso}}$ used in the calibration of the model only affects the prediction of the quantities related to the microscale (i.e., *μ*^0^ and *μ*^1^), but not the tension *T*_a_. Therefore, as far as the modeling of *T*_a_ is concerned, the model is fully characterized by the four quantities $T_{\text {a}}^{\text {iso}}$, $v^{\max \limits }$, *v*^0^ and $\tilde {k}_{2}$.

#### *Remark 2*

The relationship between the model parameters and the experimentally measured quantities listed in this Section allow to perform *sensitivity analysis* in a straightforward manner. Indeed, they provide in closed form the relationship between the parameters and the main quantities characterizing the outputs of the model. For instance, it is apparent that the only role of *a*_XB_ is that of affecting in a multiplicative manner the predicted tension, while it leaves the shape of the force-velocity curve and that of the fast transient response unaffected. On the other hand, the rate *r*_0_ is inversely proportional to the isometric tension $T_{\text {a}}^{\text {iso}}$ and to the characteristic velocities $v^{\max \limits }$ and *v*^0^, while it does not affect the shape of the fast transient response curve. Similar considerations can be done for the other parameters.

#### *Remark 3*

From a dimensional viewpoint, the parameter *a*_XB_ has the dimension of a pressure (force per unit surface), the three parameters $\mu _{\bar {f}}^{0}$, $\mu _{\bar {f}}^{1}$ and *r*_0_ have the dimension of inverse of time constants, whereas *α* is dimensionless. Hence, if we normalize pressures by *a*_XB_ and time by $r_{0}^{-1}$, it turns out the dimensionless form of the model considered in this section depends on the three dimensionless quantities $\mu _{\bar {f}}^{0}/r_{0}$, $\mu _{\bar {f}}^{1}/r_{0}$ and *α*.

### Numerical Results

In this section, we perform the calibration of the parameters of model (17), by using the relationships derived in Section 4.5 ((21) and (22)), starting from experimental measurements, reported in Table 1, together with a reference to the source in the literature. We consider data coming from intact (i.e., non skinned, see [2, 21, 26, 49]) cardiac rat cell at room temperature. The unique datum not satisfying these conditions is $\mu ^{0}_{\text {iso}}$ (which is acquired from skeletal frog muscle). However, as we mentioned in Section 4.5, the value of such parameter only affects the value of the microscopic variables (i.e., *μ*^*p*^), but not the predicted active tension. In Table 2 we report the parameters obtained by calibrating the model in both the sublinear and linear growth cases.

**Table 1:** List of the experimental data used for model calibration

Parameter	Value	Units	Reference
$T_{\text {a}}^{\text {iso}}$	120	kPa	[80]
$\mu ^{0}_{\text {iso}}$	0.22	-	[6]
$v^{\max \limits }$	8	s^− 1^	[9]
*v*^0^	2	s^− 1^	[9]
$\tilde {k}_{2}$	66	-	[9]

**Table 2:** List of the calibrated parameters in the sublinear, linear and superlinear growth cases

Parameter	Units	Sublinear growth	Linear growth	Superlinear growth
*a*_XB_	MPa	35.46	22.16	20.46
$\mu _{\bar {f}}^{1}$	s^− 1^	0.7040	0.7040	0.7040
$\mu _{\bar {f}}^{0}$	s^− 1^	45.76	28.60	2.640
*α*	−	39.00	24.37	2.250
*r*_0_	s^− 1^	208.0	130.0	12.00

**Fig. 9:**
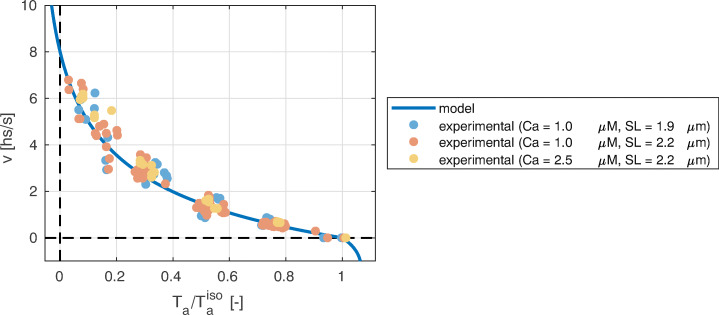
Force-velocity relationship obtained with the model (17), compared with experimental data from [9]

In Fig. 9 we show the force-velocity relationship obtained with the calibrated model (in the linear growth case), together with the experimental data from [9]. We consider only the linear growth case because the force-velocity curve is affected just by the values taken by *q*(*v*) for velocities smaller than $v^{\max \limits }$ and, hence, it does not depend on the asymptotic behavior of *q*(*v*).We can see that the calibration procedure is successful, as there is a good match between the prediction of the model and the experimental measurements.

**Fig. 10:**
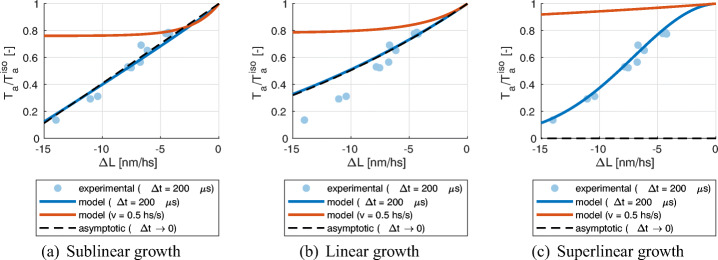
Normalized force after the application of a fast length step Δ*L*. The fast steps reported by the blue lines (model result) and the blue circles (*T*_2_-*L*_2_ experimental data from [9]) are applied within a time interval of Δ*t* = 200μs, whereas the red lines refer to fast steps applied with a shortening velocity of *v* = 0.5s^− 1^. Finally, the black dashed lines refer to the asymptotical response for Δ*t* → 0

Then, in Fig. 10 we consider the fast response predicted by the model. With this aim, we let the model reach the steady state and then we apply a length step, by applying a constant velocity in a small time interval Δ*t*. Finally, we plot the tension obtained at the end of the step against the step length Δ*L*. We repeat this protocol twice: first, by reproducing the same conditions employed in laboratory, that is by applying the length step in a very small time interval (Δ*t* = 200μs, see [9] and Section 2.3); then, we repeat the simulation, this time by applying the step with a lower shortening velocity, compatible with the typical velocity by which the cardiac tissue shortens during a heartbeat (we set *v* = 0.5s^− 1^).

We show in Figs. 10a and 10b the results obtained, in the case of sublinear (by setting $q(v) = \alpha \sqrt {|v|}$) and linear (by setting *q*(*v*) = *α*|*v*|) growth of *q*, respectively. The models here considered do not explicitly represent the power stroke, whose effect is instead accounted for in the definition of the attachment-detachment rates (see Section 4.5). Therefore, we compare the tension after the 200μs fast transient with the *T*_2_-*L*_2_ data, experimentally measured by applying a fast step within the same time interval (see again Section 4.5). The good match between the simulation results and the experimental measurements provides a further validation of the calibration procedure.

The curves obtained by letting the tissue shorten with a velocity similar to that observed during a heartbeat are close to those obtained with an almost instantaneous step, for small values of Δ*L*. Conversely, for larger Δ*L*, the former curves saturate and a smaller force drop is observed. The reason is that a large length step takes a longer time to be accomplished, and, consequently, the time interval is large enough for the attachment-detachment process to partially recover the original tension. In other terms, when we consider the typical time scales of a heartbeat, the dynamics of the length changes is not sufficiently fast to appreciate the scale separation between the different phases following a fast transient (see Section 2.3). This provides a justification for the fact that a lumped description of the power stroke is an acceptable approximation if the model is used for organ-level simulations and for the fact that, in the model calibration, fitting the *T*_2_-*L*_2_ curve for small values of Δ*L* is sufficient (see Section 4.5).

Finally, in Fig. 10c we show the fast-transient obtained in the case of superlinear growth of *r* (by setting *q*(*v*) = *α*(|*v*| + *v*^2^)). In this case, since we do not have a relationship equivalent to (21) and (22), we employ the relationship derived in the linear growth case, by adjusting the parameter $\tilde {k}_{2}$ to fit the experimental data. We notice that, even if the asymptotic analysis of Section 4.4 shows that, in the limit of $v\to \infty $, the response to fast steps leads to vanishing tension, when the step is applied within a finite time interval, we obtain a curve that is in agreement with the experimental measurements.

## Conclusions

In this paper, we reviewed several models describing the interaction between actin and myosin in cardiac muscle cells. As a matter of fact, different models, with different degrees of biophysical detail, are available in the literature. The most detailed models are able of capturing phenomena, such as the response to fast steps, occurring at the fastest time scales involved in the force generation mechanism [12, 55, 56]. Conversely, the models belonging to the family of the H57 model, while being able to reproduce the phenomena occurring at slower time scales (such as the force-velocity relationship), do not allow to match the two different experimentally observed fast responses exhibited by the muscle tissue when a step (either in length or in tension) is applied.

In [12] the authors show that, if the considered time scales are large enough for the variable describing the power stroke to be considered at thermal equilibrium, detailed soft-spring models that explicitly represent the power stroke are formally recast to the H57 model. Motivated by this observation, we have investigated the capabilities of a modified version of the H57 model to reproduce the experimentally observed characterizations of the force generation phenomenon. Such model, compared to the most detailed models that explicitly represent the power stroke, has the significant advantage of featuring only five parameters (of which four are independent parameters for the determination of the active tension *T*_a_), that can be determined starting from macroscopic measurements typically available from experiments. In particular, the model can match the isometric active tension, the force-velocity relationship and the stiffness associated to small steps. Hence, if the characteristic time scales of the phenomena under exam are slower than the fast time scale of the power stroke (such as in full-organ cardiac simulations), the models of the H57 family match a good balance between model accuracy and parameter identifiability. On the other hand, the limitations of the models of this family show up when faster times scales are addressed. In particular, they are not able to predict the observed spontaneous oscillations that can be reproduced by models that, on the contrary, explicitly represent the power stroke (see e.g. [84]). Finally, a limitation of the modified H57 model considered in this paper is to ascribe to the dependence of the detachment rate on the shortening velocity for which, to the best of our knowledge, experimental validation is still missing.
